# Antibodies Against β_2_-Glycoprotein I Complexed With an Oxidised Lipoprotein Relate to Intima Thickening of Carotid Arteries in Primary Antiphospholipid Syndrome

**DOI:** 10.1080/17402520600554930

**Published:** 2006-03

**Authors:** P. R. J. Ames, J. Delgado Alves, L. R. Lopez, F. Gentile, A. Margarita, L. Pizzella, J. Batuca, G. Scenna, V. Brancaccio, E. Matsuura

**Affiliations:** 1 Academic Department of Rheumatology Leeds General Infirmary Leeds UK; 2 Department of Pharmacology Faculty of Medical Sciences New University of Lisbon Lisbon Portugal; 3 Corgenix, Inc. Westminster Colorado USA; 4 Department of Health Sciences (SpeS) Molise University Campobasso Italy; 5 Angiology and Coagulation Units Cardarelli Hospital Naples Italy; 6 Department of Animal, Agricultural & Environmental Sciences (SAVA) Molise University Campobasso Italy; 7 Department of Cell Chemistry Graduate School of Medicine Dentistry and Pharmaceutical Sciences Okayama University Okayama Japan

## Abstract

To explore whether antibodies against β_2_-glycoprotein I (β_2_GPI) complexed to 7-ketocholesteryl-9-carboxynonanoate (oxLig-1) and to oxidised low-density lipoproteins (oxLDL) relate to paraoxonase activity (PONa) and/or intima media thickness (IMT) of carotid arteries in primary antiphospholipid syndrome (PAPS). As many as 29 thrombotic patients with PAPS, 10 subjects with idiopathic antiphospholipid antibodies (aPL) without thrombosis, 17 thrombotic patients with inherited thrombophilia and 23 healthy controls were investigated. The following were measured in all participants: β_2_GPI−oxLDL complexes, IgG anti-β_2_GPI−oxLig-1, IgG anti-β_2_GPI−oxLDL antibodies (ELISA), PONa, (para-nitrophenol method), IMT of common carotid (CC) artery, carotid bifurcation (B), internal carotid (IC) by high resolution sonography. β_2_GPI−oxLDL complex was highest in the control group (*p* < 0.01), whereas, IgG anti-β_2_GPI−oxLig1 and IgG anti-β_2_GPI−oxLDL were highest in PAPS (*p* < 0.0001). In healthy controls, β_2_GPI−oxLDL complexes positively correlated to IMT of the IC (*p* = 0.007) and negatively to PONa after correction for age (*p* < 0.03). PONa inversely correlated with age (*p* = 0.008). In PAPS, IgG anti-2GPI−oxLig-1 independently predicted PONa (*p* = 0.02) and IMT of B (*p* = 0.003), CC, (*p* = 0.03) and of IC (*p* = 0.04). In PAPS, PONa inversely correlated to the IMT of B, CC and IC (*p* = 0.01, 0.02 and 0.003, respectively). IgG anti-2GPI−oxLig-1 may be involved in PAPS related atherogenesis via decreased PON activity.


*Clinical & Developmental Immunology*, March 2006; 13(1): 1–9

Taylor & Francis

Taylor & Francis Group

### Antibodies against $\beta_2$-glycoprotein I complexed with an oxidised lipoprotein relate to intima thickening of carotid arteries in primary antiphospholipid syndrome

P.R.J. AMES[1], J. DELGADO ALVES[2], L.R. LOPEZ[3], F. GENTILE[4], A. MARGARITA[5], L. PIZZELLA[6], J. BATUCA[2], G. SCENNA[5], V. BRANCACCIO[5], & E. MATSUURA[7]

[1]*Academic Department of Rheumatology, Leeds General Infirmary, Leeds, UK,* [2]*Department of Pharmacology, Faculty of Medical Sciences, New University of Lisbon, Lisbon, Portugal,* [3]*Corgenix, Inc, Westminster, Colorado, USA,* [4]*Department of Health Sciences (SpeS), Molise University, Campobasso, Italy,* [5]*Angiology and Coagulation Units Cardarelli Hospital, Naples, Italy,* [6]*Department of Animal, Agricultural & Environmental Sciences (SAVA), Molise University, Campobasso, Italy, and* [7]*Department of Cell Chemistry, Graduate School of Medicine, Dentistry and Pharmaceutical Sciences, Okayama University, Okayama, Japan*

**Abstract**
To explore whether antibodies against $\beta_2$-glycoprotein I ($\beta_2$GPI) complexed to 7-ketocholesteryl-9-carboxynonanoate (oxLig-1) and to oxidised low-density lipoproteins (oxLDL) relate to paraoxonase activity (PONa) and/or intima media thickness (IMT) of carotid arteries in primary antiphospholipid syndrome (PAPS). As many as 29 thrombotic patients with PAPS, 10 subjects with idiopathic antiphospholipid antibodies (aPL) without thrombosis, 17 thrombotic patients with inherited thrombophilia and 23 healthy controls were investigated. The following were measured in all participants: $\beta_2$GPI–oxLDL complexes, IgG anti-$\beta_2$GPI–oxLig-1, IgG anti-$\beta_2$GPI–oxLDL antibodies (ELISA), PONa, (para-nitrophenol method), IMT of common carotid (CC) artery, carotid bifurcation (B), internal carotid (IC) by high resolution sonography. $\beta_2$GPI–oxLDL complex was highest in the control group ($p < 0.01$), whereas, IgG anti-$\beta_2$GPI–oxLig1 and IgG anti-$\beta_2$GPI–oxLDL were highest in PAPS ($p < 0.0001$). In healthy controls, $\beta_2$GPI–oxLDL complexes positively correlated to IMT of the IC ($p = 0.007$) and negatively to PONa after correction for age ($p < 0.03$). PONa inversely correlated with age ($p = 0.008$). In PAPS, IgG anti-$\beta_2$GPI–oxLig-1 independently predicted PONa ($p = 0.02$) and IMT of B ($p = 0.003$), CC, ($p = 0.03$) and of IC ($p = 0.04$). In PAPS, PONa inversely correlated to the IMT of B, CC and IC ($p = 0.01$, 0.02 and 0.003, respectively). IgG anti-$\beta_2$GPI–oxLig-1 may be involved in PAPS related atherogenesis via decreased PON activity.

**Keywords:** *Carotid arteries, oxidation, primary antiphospholipid syndrome*

### Introduction

Thrombosis is the main vascular feature of the primary antiphospholipid syndrome (PAPS), but some evidence suggests that vascular damage in PAPS may include also atherosclerosis (Ames et al. 2005). Most of the human data relating antiphospholipid antibodies (aPL) to atherosclerosis derive from studies on APS related to systemic lupus erythematosus, where aPL are variably associated with greater intima-media thickness (IMT) and/or focal plaques of carotid arteries although not predictive of atherosclerosis in regression models (Manzi et al. 1999, Asanuma et al. 2003, Roman et al. 2003). Nevertheless, mice immunised with aPL and with $\beta_2$GPI, the target antigen of aPL, develop atherosclerosis (George et al. 1998) and aPL titre independently predicted IMT of carotid arteries, an acceptable and reliable surrogate marker of sub-clinical atherosclerosis, in patients with PAPS (Ames et al. 2002).

An imbalance between oxidative stress and antioxidant defence contributes to atherogenesis. We previously

Correspondence: P. R. J. Ames, Academic Department of Rheumatology, Leeds General Infirmary, Great George Street, Leeds LS1 3EX, UK. E-mail: paxmes@aol.com

ISSN 1740-2522 print/ISSN 1740-2530 online © 2006 Taylor & Francis
DOI: 10.1080/17402520600554930

demonstrated enhanced oxidative stress (Ames et al. 1998) as well as lower antioxidant defence measured as decreased paraoxonase activity (PONa) in PAPS (Delgado Alves et al. 2002). PON is the enzyme that accounts for most of the capacity of high-density lipoprotein to degrade oxidised phospholipids into inactive metabolites minimizing the chance of low-density lipoprotein (LDL) oxidation (Leviev and James 2000). Of further interest is the knowledge that $\beta_2$GPI binds to $Cu^{2+}$–oxidised LDL (oxLDL) (Kobayashi et al. 2003) and that macrophages uptake this complex much faster in the presence of an antibody towards oxLDL–$\beta_2$GPI (Hasunuma et al. 1997). More recently, this concept has been extended to the complex of $\beta_2$GPI with 7-ketocholesteryl-9-carboxynonanoate (oxLig-1), the very oxLDL-derived moiety that binds $\beta_2$GPI (Liu et al. 2002). As antibodies towards $\beta_2$GPI–oxLig-1 are associated with arterial thrombosis in APS (Lopez et al. 2003) we herein explored whether antibodies towards anti $\beta_2$GPI–oxLig-1 bore any atherogenic potential in primary APS.

### Methods

### *Patients*

The study populations were drawn from consecutive patients attending the Coagulation Unit of the Cardarelli Hospital in Naples (Italy) who gave informed consent before entering the study, carried out according to the revised Declaration of Helsinki. All APS patients met the Sapporo Criteria for PAPS (Wilson et al. 1999). They were evaluated for a thrombotic event and were found to be positive for lupus anticoagulant (LA) and anticardiolipin (aCL) antibodies on two separate occasions 6 weeks apart. Patients with idiopathic aPL were detected because of the presence of a LA on routine clotting screen for minor surgical procedures or for health checks and never suffered any vascular manifestation of APS. Seventeen patients with inherited thrombophilia who underwent vascular occlusions served as a thrombotic control group. Vascular events were diagnosed by MRI, doppler ultrasound, ECG and echocardiogram according to vascular bed involved. Twenty-three healthy hospital personnel served as a normal control group. None of the participants were on lipid lowering agents or antioxidant drugs at the time of the study. Plasma from patients and controls were kept at 80°C until analysis. Their demographics and clinical features are presented in Table I.

### *Antiphospholipid antibodies*

IgG aCL antibodies were measured by ELISA (Cambridge Life Sciences, UK). A normal range had been determined from 60 healthy hospital personnel with a cut-off of 5 GPL U/ml being five standard errors above the geometric mean (4.1; SEM 0.2; 95% CI 3.7, 4.5). IgG anti-$\beta_2$GPI antibodies were measured by ELISA (Corgenix, Westminster, Co.). As many as 120 serum samples from healthy blood bank donors were tested for IgG anti-$\beta_2$GPI antibodies to establish the cut-off of the assay at 20 units. Intra and inter assay coefficient of variability was 4.1 and 3%, respectively. LA was screened by activated partial thromboplastin time, dilute Russel viper venom time and kaolin clotting time. After detecting an inhibitor by mixing studies, the platelet neutralisation procedure in the activated partial thromboplastin time and dilute Russel viper venom time was used to confirm the presence of a LA in accordance to published guidelines (Greaves et al. 2000).

### *Monoclonal antibodies*

The following monoclonal antibodies were employed as described in the assays reported below. WB-CAL-1 is an IgG2a murine monoclonal antibody against human $\beta_2$GPI derived from a NZW × BXSB F1 mouse, a model of spontaneous APS (Hashimoto et al. 1992). EY2C9 is a human monoclonal IgM anti-$\beta_2$GPI antibody established from peripheral blood lymphocytes of a patient with APS (Ichikawa et al. 1994). These two antibodies bind only to $\beta_2$GPI-negatively charged phospholipid (or oxLDL) complexes but not with monomeric (free) $\beta_2$GPI in solution. ID2 is a murine IgG monoclonal antibody specific for human apolipoprotein B-100 that reacts equally well with native and oxLDL. IS-4 is another human monoclonal IgG anticardiolipin established from peripheral blood lymphocytes of patients with APS (Zhu et al. 1999).

### *Measurement of $\beta_2$GPI–oxLDL complexes*

The monoclonal antibody against $\beta_2$GPI (WB-CAL-1) was adsorbed on a microtiter plate (Immulon 2HB, Dynex Technology, Inc., Chantilly, VA) by incubating overnight 50 μl/well at a concentration of 50 μl/ml dissolved in PBS buffer, pH 7.4, between 2 and 4°C. In this assay, WB-CAL-1 captures $\beta_2$GPI–oxLDL complexes via its specificity for $\beta_2$GPI. The plate was blocked with PBS-1% skim milk for 1 h. Then, 100 μl of samples diluted 1:25 in PBS skim milk containing 10 m mol/l $MgCl_2$ were added to the wells and incubated for 2 h at room temperature. $MgCl_2$ dissociates intermediate $\beta_2$GPI–oxLDL complexes bound via electrostatic forces allowing the specific detection of non-dissociable and covalently bound complexes present in plasma samples (Kobayashi et al. 2003). The wells were washed 4-fold with PBS-0.05% polysorbate 20 between each step then incubated with biotinyl-anti-apoB-100-Ab (1D2) diluted in PBS skim milk for 1 h at room temperature. Horseradish peroxidase streptavidin was added for 30 min, colour was developed with tetramethylbenzidine-$H_2O_2$, and

Table I. Demographics and clinical features of the study populations.

| | CTR | THR CTR | APL | PAPS |
|---|---|---|---|---|
| *n* | 23 | 17 | 10 | 29 |
| Female/male | 13/7 | 11/6 | 7/3 | 20/9 |
| Age (median, range) | 35 (20–68) | 40 (30–58) | 40 (29–54) | 38 (27–63) |
| IgG aCL (median, range) | | | 59 (16–309) | 118 (24–573) |
| IgG anti-$\beta_2$GPI | | | 147 (40–216) | 183 (1–226) |
| FVL | 0 | 12 | 0 | 2 |
| PT 20210 | 1 | 2 | 1 | 1 |
| PC deficiency | 0 | 3 | 0 | 0 |
| IS | | 2 | | 8 |
| MI | | 0 | | 1 |
| DVT | | 11 | | 15 |
| PE | | 4 | | 5 |
| Smoking | 3 | 4 | 2 | 5 |
| Hypertension | 0 | 0 | 0 | 1 |
| Diabetes | 0 | 0 | 0 | 1 |
| Obesity | 0 | 0 | 0 | 0 |
| High cholesterol | 0 | 0 | 0 | 1 |
| High triglycerides | 0 | 0 | 1 | 2 |
| Aspirin (75 mg) | 0 | 1 | 6 | 0 |
| Warfarin | 0 | 17 | 0 | 29 |

CTR: controls; THR CTR: thrombotic controls; APL: antiphospholipid antibodies positive without thrombosis and underlying disorder; PAPS: primary antiphospholipid syndrome; FVL: factor V Leiden; PT: prothrombin 20210; PC: protein C; IS: ischaemic stroke; MI: myocardial infarction; DVT: deep vein thrombosis; PE: pulmonary embolism.

the reaction stopped with 0.36 N sulphuric acid. Optical density was read at 450 nm (650 nm reference). The coefficient of variation ranged between 7.2 and 12.3% for weak and 4.5–8.9% for moderate and strong reactive samples. The concentration of $\beta_2$GPI–oxLDL complexes in U/ml was calculated against a reference curve built with 2-fold serial dilutions of a $\beta_2$GPI–oxLDL complex solution. The complexes were prepared in advance by incubating equal amounts of $Cu^{2+}$–oxLDL and purified human $\beta_2$GPI, pH 7.4, for 12 h at 37°C. The unit value derives arbitrarily from the protein concentration of the $\beta_2$GPI–oxLDL used in the reference curve. A normal cut-off value for the assay was established testing samples from 100 healthy blood donors (mean + 3SD).

### *Measurement of IgG anti-$\beta_2$GPI–oxLig-1 antibodies*

OxLig-1 (7-ketocholesteryl-9-carboxynonanoate) is a $Cu^{2+}$-derived LDL ligand for $\beta_2$GPI prepared as previously described (Liu et al. 2002). Fifty microliter of 100 μg/ml of oxLig-1 in ethanol were adsorbed by evaporation on a plain polystyrene plate (Immulon 2HB) followed by blocking with 1% BSA for 1 h at room temperature and then washed. Subsequently, 50 μl of 30 μg/ml of $\beta_2$GPI in PBS with 3% BSA was added to oxLig-1-coated wells to allow complex formation. The $\beta_2$GPI–oxLig-1 complex represents the capture antigen for patient antibodies. Finally, 50 μl of plasma samples diluted 1:100 in PBS with 3% BSA were added to the wells and incubated for 1 h at room temperature. Wells were washed four times with PBS 0.05% polysorbate 20 between steps. Diluted HRP-conjugated antihuman IgG was added and left to incubate for 1 h. Colour was developed with tetramethylbenzidine-$H_2O_2$, and the reaction stopped with 0.36 N sulphuric acid. Optical density was read at 450 nm (650 nm reference). The coefficient of variation ranged between 7.4 and 12.6% for weak and 5.5–9.9% for moderate and strong reactive samples. The EY2C9 IgM monoclonal anti-$\beta_2$GPI was used in this assay as a reference human antibody reacting to the $\beta_2$GPI of the antigenic substrate to select strong reactive samples to be used as control samples. However, the IgG $\beta_2$GPI–oxLig-1 concentration of patient samples (in U/ml) was calculated against a standard curve prepared from a selected positive sample. A normal cut-off value for the assay was established by testing 100 serum samples from healthy blood donors (mean + 3SD).

### *Measurement of IgG anti-$\beta_2$GPI–oxLDL antibodies*

LDL was isolated and oxidised as previously described (Kobayashi et al. 2003). OxLDL (50 μg/ml in ethanol, 50 μl well) was adsorbed by evaporation on plain polystyrene plates (Immulon 1B) and the plates blocked with 1% BSA. Serum samples (diluted 1:100) were incubated in the wells with or without $\beta_2$GPI (25 μg/ml) for 1 h followed by the addition of HRP-labelled antihuman IgG. Further steps were performed as in ELISA for anti-$\beta_2$GPI–oxLig-1. The mean OD of the blank wells was employed to correct the raw OD of individual samples. OD variation among plates was normalised using a positive control. A sample was considered to be positive when its antibody titre was higher than three standard deviations above the mean OD of plasma samples from 150 normal subjects (blood donors).

*Paraoxonase activity*

Serum PON activity was measured as previously described (Delgado Alves et al. 2002). Briefly, PON (1.0 mM) freshly prepared in 300 μl of 50 mM glycine buffer containing 1 mM calcium chloride (pH 10.5) was incubated at 37°C with 5 μl of serum for 15 min in 96 well plates (Polysorp, Nunc, Life Technologies, Paisley, UK). Para-nitrophenol formation was monitored at 412 nm and activity expressed as nmol of para-nitrophenol per ml serum per minute. For graphical purposes baseline PONa activity was set at 100% with percentage decrements thereafter.

*Effect of monoclonal antiphospholipid antibodies on paraoxonase activity*

The possible inhibitory effect of aPL on PON activity was explored. Dose dependency was performed by incubating doubling dilutions (2, 4, 8 and 16 fold) of IS4 supernatant (starting at 10 g/ml, the maximum supernatant concentration), WB-CAL-1 and EY2C9 with 50 μl of pooled sera from five healthy subjects as a source of PON. PON activity was then measured every 30 min for 4.5 h, because specific paraoxon hydrolysis starts at about 2 h from its incubation with PON. As controls we used irrelevant human IgG (Sigma-Aldrich, Poole, UK) at the same concentration. The possible effect of $\beta_2$GPI and $\beta_2$GPI–oxLDL on PON was also investigated. All experiments were run in triplicate.

*Carotid intima-media thickness measurement*

All participants underwent a detailed ultrasound evaluation of the carotid arteries performed by an experienced angiologist (AM), using an Aloka 2000 sonograph equipped with a 5–10 MHz linear transducer. The image acquisition followed the Atherosclerosis Risk in Communities Study protocol (Heiss et al. 1991). Images were obtained with the patient in the supine position with the neck slightly extended. Transverse and longitudinal views of the right and left common carotid (CC) arteries (10 mm distal to the carotid bifurcation), right and left carotid bifurcation (B) and internal carotid (IC) arteries were taken. The means of three measurements taken 1 cm apart at the three different sites were computed for each region, right and left measurements were averaged for the purpose of statistical analysis. Plaques, defined as the presence of focal, severe wall thickening (IMT > 2 mm), wall irregularities and calcification, were also looked for. Intra-reader reproducibility was assessed in two ways. Firstly, the same carotid measurement was performed twice on 20 individuals yielding a coefficient of agreement of 97%. Secondly, the same IMT measurements were performed six times on one individual over a two-month period, yielding a coefficient of variability between 3 and 4% according to carotid segment under study.

*Statistics*

Non-parametric tests were employed throughout because of the small numbers in groups. Comparisons across groups were assessed by Kruskall–Wallis analysis of variance with Dunns *post hoc* as appropriate. Univariate relationships between variables were examined by Spearman rank and implemented where described by multiple regression analysis.

### Results

*PON activity, **$\beta_2$**GPI–oxLDL complexes, anti-**$\beta_2$**GPI–oxLDL and anti-**$\beta_2$**GPI–oxLig-1 in study groups*

PON activity (n mol/ml/min) was not different across the groups, being 0.96 ± 0.25 in normal controls, 0.90 ± 0.340 in thrombotic controls, 0.87 ± 0.40 in idiopathic aPL and 78 ± 0.35 in APS patients. Plasma levels of $\beta_2$GPI–oxLDL complexes were highest in the control group (Figure 1A), whereas, those of IgG anti-$\beta_2$GPI–oxLDL (Figure 1B) and anti-$\beta_2$GPI–oxLig-1 (Figure 1C) were highest in the APS and aPL groups. These variables showed no gender differences in any of the groups, and no differences were noted between patients with arterial or venous thrombosis in the PAPS group.

*Relationship amongst variables in the normal control group*

Several variables in this group were age related: PONa (Figure (2A)), $\beta_2$GPI–oxLDL complexes ($r = 0.61$, $p = 0.001$) IgG anti-$\beta_2$GPI–oxLig-1 ($r = 0.46$, $p = 0.02$) and IMT of all carotid segments ($p = 0.0001$). $\beta_2$GPI–oxLDL negatively correlated to PONa (Figure 2B), positively to the IMT of the IC artery (Figure 2C) and to IgG anti-$\beta_2$GPI–oxLig-1 ($r = 0.51$, $p = 0.01$). After correction for age, $\beta_2$GPI–oxLDL maintained its correlation with PONa only ($r = -0.46$, $p = 0.03$).

*Relationship amongst variables in the thrombotic control group*

$\beta_2$GPI–oxLDL complexes correlated to the IMT of the common ($r = 0.54$, $p = 0.02$) and the IC artery ($r = 0.53$, $p = 0.03$) and negatively to PONa ($r = -0.51$, $p = 0.03$). Only a trend was seen with age ($r = 0.47$, $p = 0.07$), IMT of all carotid segments strongly correlated to age ($p = 0.0001$).

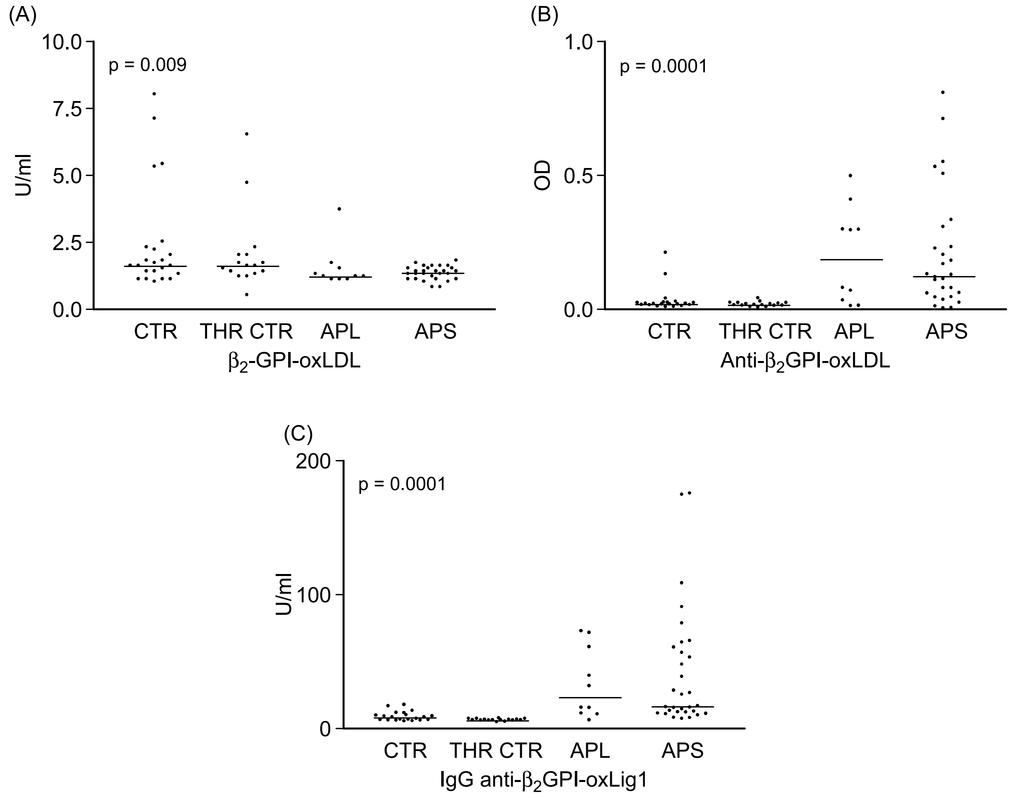

Figure 1. Median plasma levels of $\beta_2$GPI–oxLDL (A), of IgG anti-$\beta_2$GPI–oxLDL (B) and of anti-$\beta_2$GPI–oxLig-1 (C) in the four study groups. CTR: normal controls; THR CTR; thrombotic controls; aPL: non-thrombotic idiopathic antiphospholipid antibody subjects; APS: thrombotic primary antiphospholipid antibody syndrome patients.

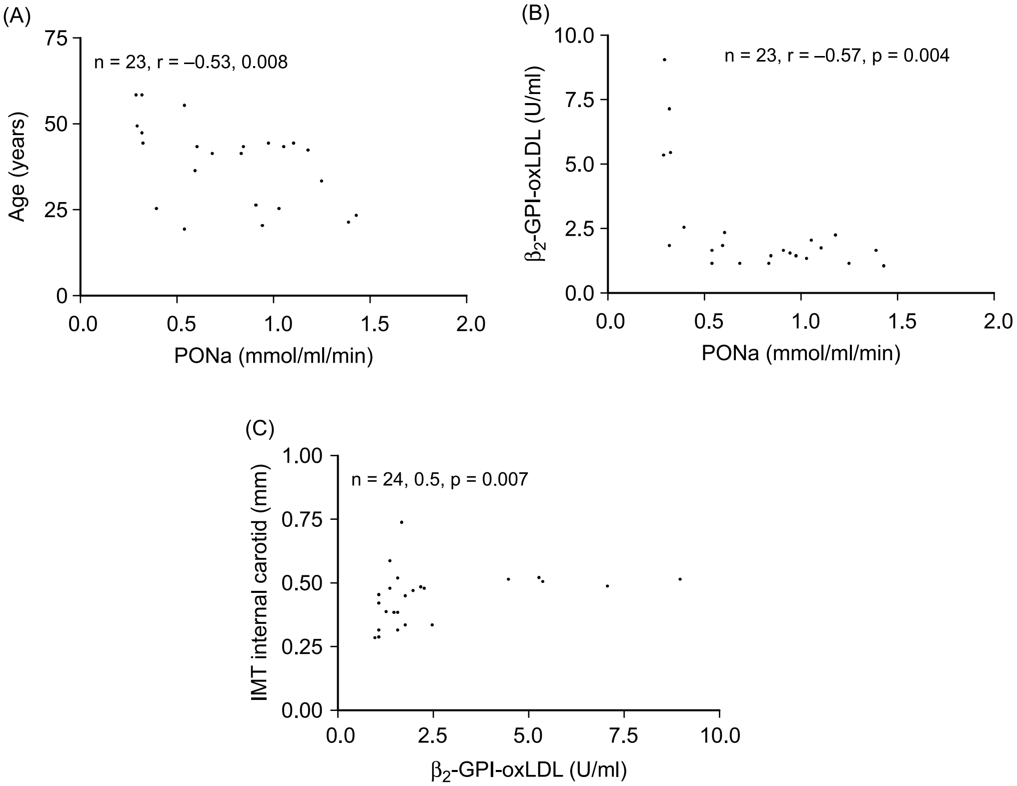

Figure 2. Relationship between PON activity and age (A), and between $\beta_2$GPI–oxLDL and PONa (B) and IMT (C) of IC arteries in normal controls.

*Relationship amongst variables in the aPL group*

IgG anti-$\beta_2$GPI–oxLig-1 correlated to the IMT of the B ($r = 0.8$, $p = 0.002$) and a trend was noted for the CC ($r = 0.52$, $p = 0.08$). No other correlations were noted because of the limited size of this group.

*Relationship amongst variables in the APS group*

Because two patients in this group did not have all variables measured, resulting analysis and data refer to 28 patients. With regards to antibody subtypes IgG anti-$\beta_2$GPI–oxLig-1 positively correlated to IgG anti-$\beta_2$GPI ($r = 0.49$, $p = 0.007$) and to IgG aCL ($r = 0.61$, $p = 0.0005$). In decreasing order of strength PONa inversely correlated to IgG anti-$\beta_2$GPI–oxLig-1 ($r = -0.51$, $p = 0.004$), to IgG aCL ($r = -0.53$, $p = 0.003$) and to IgG anti-$\beta_2$GPI ($r = -0.41$, $p = 0.02$). Of these, only IgG anti-$\beta_2$GPI–oxLig-1 independently predicted PONa ($\beta = 0.44$, $p = 0.02$) in a multiple regression that took into account the effect of age and sex. In addition, PONa inversely correlated to the IMT of the IC (Figure 3A) and of the B (Figure 3B). IgG anti-$\beta_2$GPI–oxLig-1 positively correlated to IMT of all carotid segments (Figure 4A–C). In a multiple regression model that included IgG anti-$\beta_2$GPI–oxLig-1, IgG anti-$\beta_2$GPI, and IgG anti-$\beta_2$GPI–oxLDL as independent variables and IMT as a dependent variable, and after correction for age and sex, IgG anti-$\beta_2$GPI–oxLig-1 independently predicted IMT of B ($\beta = 0.40$, $p = 0.01$), CC ($\beta = 0.36$, $p = 0.02$) and IC ($\beta = 0.55$, $p = 0.003$).

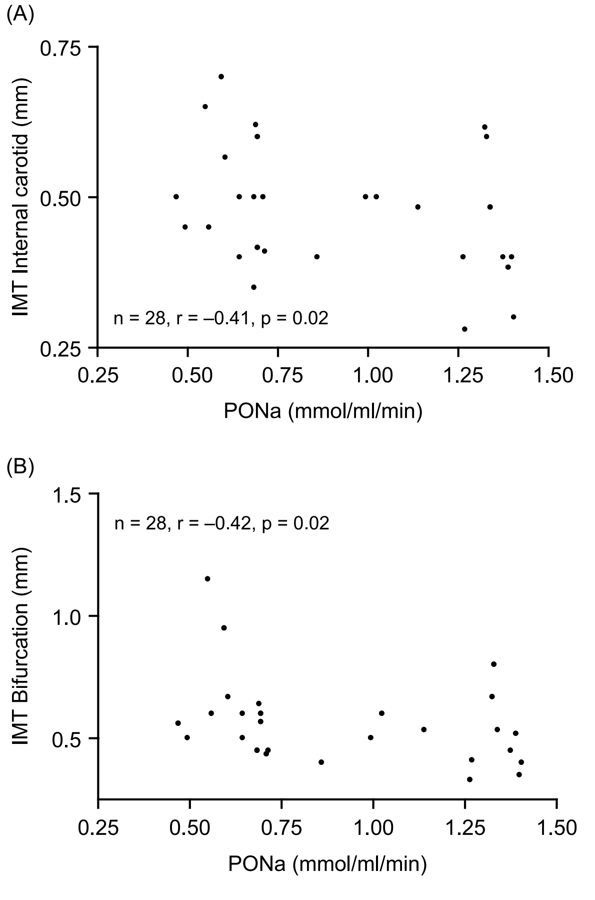

Figure 3. Relationship between PON activity and IMT of IC (A) and B (B) in PAPS.

*Effect of antiphospholipid antibodies on paraoxonase activity*

PON activity in the presence of IS4 was significantly reduced after 3 h of incubation at the starting concentration of 10 g/ml compared with the same concentration of irrelevant human IgG or PBS ($p < 0.001$)(Figure 4). WB-CAL-1 (the murine IgG antihuman $\beta_2$GPI) and EY2C9 (the human IgM anti-$\beta_2$GPI) had no effect at any concentration on PON nor did $\beta_2$GPI and $\beta_2$GPI–oxLDL complexes at any concentration (Figure 5). In dose-dependency experiments IgG aCL (IS4) significantly inhibited PON activity at the starting concentration of 10 g/ml and at 1:2 and 1:4 dilutions compared to 1:8 and 1:16 dilutions ($p < 0.001$, $<0.001$ and $<0.01$, respectively).

### Discussion

Oxidation of LDL plays an important role in atherosclerosis, as oxLDL may induce vasoconstriction, expression of adhesion molecules and cellular proliferation (Kugiyama et al. 1990, Lehr et al. 1991, Sakai et al. 1994). On the other hand, immunisation of hypercholesterolemic rabbits with oxLDL suppresses atherosclerosis (Palinski et al. 1995) therefore an immune response to oxidised LDL (oxLDL) may modulate atherogenesis (Horkko et al. 2000). An inverse relationship between oxLDL and IgG anti–oxLDL has been noted in the general population, and it was implied that anti-oxLDL helps to remove oxLDL from the bloodstream (Shoji et al. 2001). However, data contrast on this issue. One study described an inverse and independent relationship between anti-oxLDL titre and IMT of carotid arteries in healthy subjects (Fukumoto et al. 2000), whereas, IgG–oxLDL titre positively associated with IMT of the CC artery in another study (Hulthe et al. 2001). That is, both the antigen and the corresponding antibody seem to have a relation with IMT. Similar conflicting information is available for SLE, where oxLDL appeared to be related to arterial thrombosis (Amengual et al. 1997) or venous thrombosis (Hayem et al. 2001) in SLE-related APS, whereas, more recently oxLDL related to disease activity (Gomez-Zumaquero et al. 2004).

The approach used in the present study is a step ahead of the oxLDL/anti-oxLDL system. $\beta_2$GPI complexes with oxLDL and more specifically with the 7-ketocholesteryl-9-carboxynonanoate (oxLig-1) moieties from oxLDL and antibodies against the latter complex, IgG anti-$\beta_2$GPI–oxLig-1, identified arterial disease in SLE related APS (Lopez et al. 2003). The relation of this antigen/antibody system with IMT of

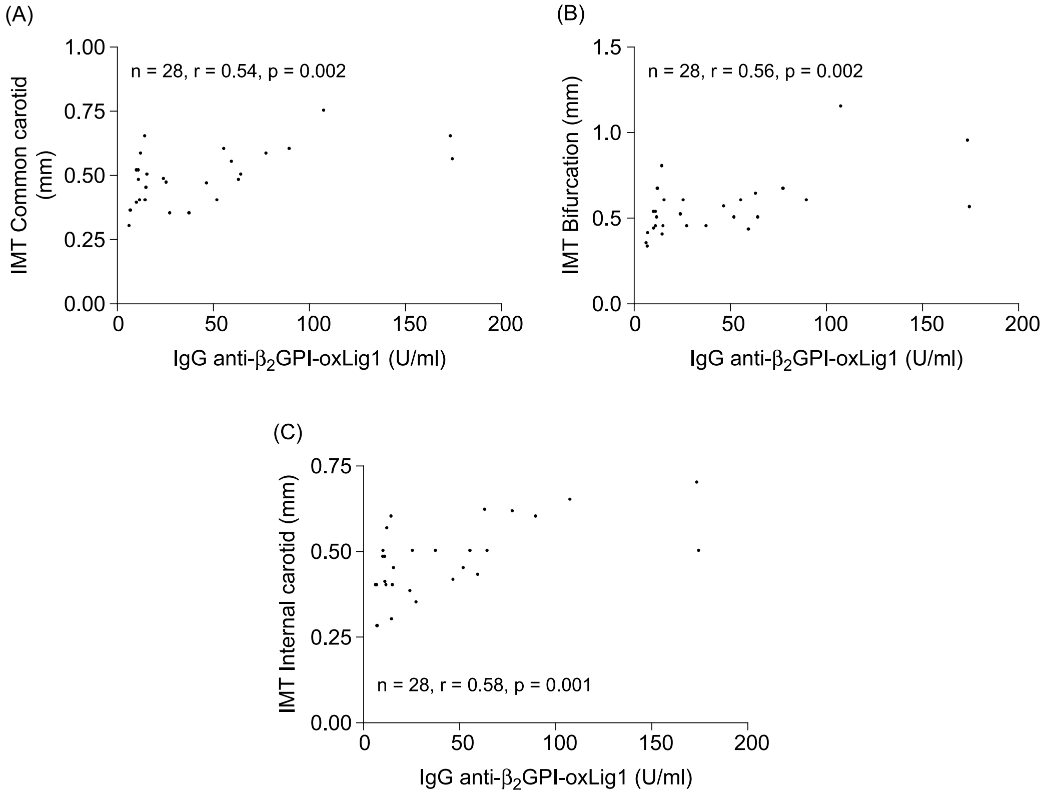

Figure 4. Relationship between IgG anti-$\beta_2$GPI–oxLig-1 and IMT of CC (A), B (B) and IC in PAPS.

carotid arteries or PON has been investigated neither in PAPS nor in a normal population.

Median plasma levels of the antigen, $\beta_2$GPI–oxLDL complexes, were higher in our control populations and lower in antiphospholipid subjects, whereas, the antibodies IgG anti-$\beta_2$GPI–oxLDL and IgG anti-$\beta_2$GPI–oxLig-1 were greatest in the PAPS group, though they did not identify patients with arterial thrombosis. With regards to PONa we did not confirm our previous report of low activity in PAPS (Delgado Alves et al. 2002) probably due to different control populations bearing different genetic polymorphisms that partly govern the enzymatic activity of PON (Leviev and James 2000). Notwithstanding, PONa showed several interesting relationships. In the control group, $\beta_2$GPI–oxLDL inversely related to PONa, indicating that as more LDL becomes oxidised, more oxLDL is buffered by $\beta_2$GPI. This is likely due to an age related decrement of the antioxidant capacity of PON (Milochevitch and Khalil 2001) given the lack of effect of $\beta_2$GPI–oxLDL on PONa in our *in vitro* experiments and the loss of correlation between $\beta_2$GPI–oxLDL and IMT of carotid segments after correction for age. We cannot discount the possibility that IgG anti-$\beta_2$GPI–oxLig-1 appears as an attempt to clear $\beta_2$GPI–oxLDL, in a fashion similar to that described between oxLDL and IgG oxLDL in normal subjects (Shoji et al. 2001). The data of our thrombotic control group partially replicate those of our normal control group and altogether they suggest that $\beta_2$GPI–oxLDL, IgG anti-$\beta_2$GPI–oxLig-1 and anti $\beta_2$GPI–oxLDL might have a role in vascular ageing in normal people.

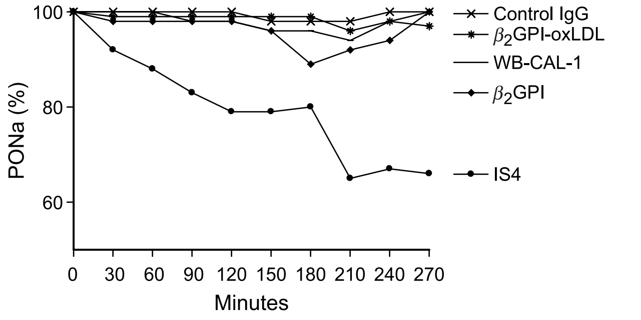

Figure 5. Effect of monoclonal antibodies IS4, WB-CAL-1 and of $\beta_2$GPI, $\beta_2$GPI–oxLDL compared to irrelevant human IgG (at 210 min IgG IS4 vs control IgG, $p < 0.001$).

The PAPS group shows picture almost specular to that of the control groups. In fact, PONa negatively related to IgG $\beta_2$GPI–oxLig-1, to IgG $\beta_2$GPI and IgG aCL, in keeping with our hypothesis that PONa inhibition participates in APS related vascular damage (8, 32). Here, we extend this concept, in that elevated IgG anti-$\beta_2$GPI–oxLig-1 together with low PONa were positive and negative correlates of the IMT of carotid artery segments and because IgG aCL inhibited PONa *in vitro*. The latter effect was limited to IS4, the monoclonal IgG aCL, whereas, WB-CAL-1, the monoclonal IgG anti-$\beta_2$GPI was devoid of such effect.

This is in keeping with the inhibitory effects of IS4 on PONa in SCID mice, where no effect was seen for anti-$\beta_2$GPI of either isotype (of a different source from the ones used here (Delgado Alves et al. 2005)). Further studies are required to elucidate the interaction between aPL and PON.

With consideration to the small numbers of our groups, we suggest the following sequence of events. In normal people, an age related decrease of PONa allows for oxidation of LDL that is buffered by $\beta_2$GPI and eventually an antibody develops to clear this ternary complex. By contrast, in PAPS, aPL interference with PONa tilts the oxidant/antioxidant balance towards enhanced lipid peroxidation (Ames et al. 1998) that in turn contributes to arterial thickening. Although, this study was not devised to demonstrating premature vascular damage in PAPS, it provides further evidence for PON and IgG anti-$\beta_2$GPI–oxLig-1 implication in an atherogenic pathway that may represent a target for therapeutic intervention.

### References

Amengual O, Atsumi T, Khamashta MA, Tinahones F, Hughes GRV. 1997. Autoantibodies against oxidised low-density lipoprotein in antiphospholipid syndrome. Br J Rheumatol 369:964–968.

Ames PRJ, Margarita A, Delgado Alves J, Tommasino C, Iannaccone L, Brancaccio V. 2002. Anticardiolipin antibody titre and plasma homocysteine level independently predict intima media thickness of carotid arteries in subjects with idiopathic antiphospholipid antibodies. Lupus 11:208–214.

Ames PRJ, Margarita A, Sokoll KB, Iannaccone L, Brancaccio V. 2005. Atherosclerosis in primary antiphospholipid syndrome. Preliminary data. Ann Rheum Dis 64:315–317.

Ames PRJ, Nourooz-Zadeh J, Tommasino C, Alves J, Brancaccio V, Anggard EE. 1998. Oxidative stress in primary antiphospholipid syndrome. Thromb Haemos 79:447–449.

Asanuma Y, Oeser A, Shintani AK, Turner E, Olsen N, Fazio S, Linton MF, Raggi P, Stein CM. 2003. Premature coronary-artery atherosclerosis in systemic lupus erythematosus. N Engl J Med 349:2407–2415.

Delgado Alves J, Ames PRJ, Donohue S, Stanyer L, Nourooz-Zadeh J, Ravirajan C, Isenberg DA, Noorouz-Zadeh J. 2002. Antibodies to high-density lipoprotein and β-2-glycoprotein I are inversely correlated with paraoxonase activity in systemic lupus erythematosus and primary antiphospholipid syndrome. Arthritis Rheum 46:2686–2694.

Delgado Alves J, Mason LJ, Ames Paul RJ, Chen PP, Rauch J, Levine JS, Subang R, Isenberg DA. 2005. Antiphospholipid antibodies are associated with enhanced oxidative stress, decreased plasma nitric oxide and paraoxonase activity in an experimental mouse mode. Rheumatology, In press.

Fukumoto M, Shoji T, Emoto M, Kawagishi T, Okuno Y, Nishizawa Y. 2000. Antibodies against oxidised LDL and carotid artery intima-media thickness in a healthy population. Arterioscler Thromb Vasc Biol 20:703–707.

George J, Afek A, Gilburd B, Blank M, Levy Y, Aron-Maor A, Levkovitz H, Shaish A, Goldberg I, Kopolovic J, Harats D, Shoenfeld Y. 1998. Induction of early atherosclerosis in LDL-receptor-deficient mice immunized with β-2-glycoprotein I. Circulation 98:1108–1115.

Gomez-Zumaquero JM, Tinahones FJ, De Ramon E, Camps M, Garrido L, Soriguer FJ. 2004. Association of biological markers of activity of systemic lupus erythematosus with levels of anti-oxidised low-density lipoprotein antibodies. Rheumatology.

Greaves M, Cohen H, Machin SJ, Mackie I. 2000. Guidelines on the investigation and management of the antiphospholipid syndrome. Br J Haematol 109:704–715.

Hashimoto Y, Kawamura M, Ichikawa K, et al. 1992. Anticardiolipin antibodies in NZW × BXSB F1 mice: A model of antiphospholipid syndrome. J Immunol 42:1309–1311.

Hasunuma Y, Matsuura E, Makita Z, Katahira T, Nishi S, Koike T. 1997. Involvement of β 2-glycoprotein I and anticardiolipin antibodies in oxidatively modified low-density lipoprotein uptake by macrophages. Clin Exp Immunol 107:569–573.

Hayem G, Nicaise-Roland P, Palazzo E, de Bandt M, Tubach F, Weber M, Meyer O. 2001. Anti-oxidised low-density-lipoprotein (OxLDL) antibodies in systemic lupus erythematosus with and without antiphospholipid syndrome. Lupus 10:346–351.

Heiss G, Sharrett AR, Barnes R, Chambless LE, Szklo M. 1991. Alzola C for the ARIC investigators. Carotid atherosclerosis measured by B-mode ultrasound in populations: Associations with cardiovascular risk factor in the ARIC study. Am J Epidemiol 134:250–256.

Horkko S, Binder CJ, Shaw PX, Chang MK, Silverman G, Palinski W, Witztum JL. 2000. Immunological responses to oxidised LDL. Free Radic Biol Med 28:1771–1779.

Hulthe J, Bokemark L, Fagerberg B. 2001. Antibodies to oxidised LDL in relation to intima-media thickness in carotid and femoral arteries in 58-year-old subjectively clinically healthy men. Arterioscler Thromb Vasc Biol 21:101–107.

Ichikawa K, Khamashta MA, Koike T, et al. 1994. 2-glycoprotein reactivity on monoclonal anticardiolipin antibodies from patients with the antiphospholipid syndrome. Arthritis Rheum 37:1453–1461.

Kobayashi K, Kishi M, Atsumi T, Bertolaccini ML, Makino H, Sakairi N, Yamamoto I, Yasuda T, Khamashta MA, Hughes GR, Koike T, Voelker DR, Matsuura E. 2003. Circulating oxidised LDL forms complexes with β-2-glycoprotein I: Implication as an atherogenic autoantigen. J Lipid Res 44:716–726.

Kugiyama K, Kerns S, Morrisett J, Roberts R, Henry P. 1990. Impairment of endothelium-dependent arterial relaxation by lysolecithin in modified lipoproteins. Nature 344:160–162.

Lehr H, Hubner C, Nolte D, Finckh B, Biesiegel U, Kohlschutter A, Messmer K. 1991. Oxidatively modified human low-density lipoprotein stimulates leucocyte adherence onto the microvascular endothelium *in vitro*. Res Exp Med 191:85–90.

Leviev I, James RW. 2000. Promoter polymorphisms of human paraoxonase *PON1* gene and serum paraoxonase activities and concentrations. Arterioscler Thromb Vasc Biol 20:516–521.

Liu Q, Kobayashi K, Furukawa J, Inagaki J, Sakairi N, Iwado A, Yasuda T, Koike T, Voelker DR. 2002. Matsuura E Ω-carboxyl variants of 7 ketocholesteryl esters are ligands for β-2-glycoprotein I and mediate antibody-dependent uptake of oxidised LDL by macrophages. J Lipid Res 43:1486–1495.

Lopez D, Kobayashi K, Merrill JT, Matsuura E, Lopez LR. 2003. IgG autoantibodies against β-2-glicoprotein I complexed with a lipid ligand derived from oxidised low-density lipoprotein are associated with arterial thrombosis in antiphospholipid syndrome. Clin Dev Immunol 10:203–211.

Manzi S, Selzer F, Sutton-Tyrrell K, Fitzgerald SG, Rairie JE, Tracy RP, Kuller LH. 1999. Prevalence and risk factors of carotid plaque in women with systemic lupus erythematosus. Arthritis Rheum 42:51–60.

Milochevitch C, Khalil A. 2001. Study of the paraoxonase and platelet-activating factor acetylhydrolase activities with aging. Prostaglandins Leukot Essent Fatty Acids 65:241–246.

Palinski W, Miller E, Witztum JL. 1995. Immunisation of low-density lipoprotein (LDL) receptor deficient rabbits with homologous malondialdehyde-modified LDL reduces atherosclerosis. Proc Nat Acad Sci USA 92:821–825.

Roman MJ, Shanker BA, Davis A, Lockshin MD, Sammaritano L, Simantov R, Crow MK, Schwartz JE, Paget SA, Devereux RB, Salmon JE. 2003. Prevalence and correlates of accelerated atherosclerosis in systemic lupus erythematosus. N Engl J Med 349:2399–2406.

Sakai M, Miyazaki A, Hakamata H, Sasaki T, Yui S, Yamazaki M, Shichiri M, Oriuchi S. 1994. Lysophosphatidylcholine plays an essential role in the mitogenic effect of oxidised low-density lipoprotein on murine macrophages. J Biol Chem 269:31430–31435.

Shoji T, Nishizawa Y, Fukumoto M, Shimamura K, Kimura J, Kanda H, Emoto M, Kawagishi T, Morii H. 2001. Inverse relationship between circulating oxidised low density lipoprotein (oxLDL) and anti-oxLDL antibody levels in healthy subjects. Atherosclerosis 148:171–177.

Wilson WA, Gharavi AE, Koike T, et al. 1999. International consensus statement on preliminary classification criteria for definite antiphospholipid syndrome: Report of an international workshop. Arthritis Rheum 42:1309–1311.

Zhu M, Olee I, Le DT, Roubey RA, Hahn B, Woods Jr, VL, Chen PP. 1999. Characterization of IgG monoclonal anti-cardiolipin/anti-β2-GPI antibodies from two patients with antiphospholipid syndrome reveals three species of antibodies. Br J Haematol 105:102–109.

